# *In vivo *administration of BL-3050: highly stable engineered PON1-HDL complexes

**DOI:** 10.1186/1472-6904-9-18

**Published:** 2009-11-17

**Authors:** Leonid Gaidukov, Dganit Bar, Shiri Yacobson, Esmira Naftali, Olga Kaufman, Rinat Tabakman, Dan S Tawfik, Etgar Levy-Nissenbaum

**Affiliations:** 1 Department of Biological Chemistry, Weizmann Institute of Science, Rehovot 76100, Israel; 2 BioLine Innovations Jerusalem, LP, 19 Hartum Street, Jerusalem, Israel

## Abstract

**Background:**

Serum paraoxonase (PON1) is a high density lipoprotein (HDL)-associated enzyme involved in organophosphate (OP) degradation and prevention of atherosclerosis. PON1 comprises a potential candidate for *in vivo *therapeutics, as an anti-atherogenic agent, and for detoxification of pesticides and nerve agents. Because human PON1 exhibits limited stability, engineered, recombinant PON1 (rePON1) variants that were designed for higher reactivity, solubility, stability, and bacterial expression, are candidates for treatment. This work addresses the feasibility of *in vivo *administration of rePON1, and its HDL complex, as a potentially therapeutic agent dubbed BL-3050.

**Methods:**

For stability studies we applied different challenges related to the *in vivo *disfunctionalization of HDL and PON1 and tested for inactivation of PON1's activity. We applied acute, repetitive administrations of BL-3050 in mice to assess its toxicity and adverse immune responses. The *in vivo *efficacy of recombinant PON1 and BL-3050 were tested with an animal model of chlorpyrifos-oxon poisoning.

**Results:**

Inactivation studies show significantly improved *in vitro *lifespan of the engineered rePON1 relative to human PON1. Significant sequence changes relative to human PON1 might hamper the *in vivo *applicability of BL-3050 due to adverse immune responses. However, we observed no toxic effects in mice subjected to repetitive administration of BL-3050, suggesting that BL-3050 could be safely used. To further evaluate the activity of BL-3050 *in vivo*, we applied an animal model that mimics human organophosphate poisoning. In these studies, a significant advantages of rePON1 and BL-3050 (>87.5% survival versus <37.5% in the control groups) was observed. Furthermore, BL-3050 and rePON1 were superior to the conventional treatment of atropine-2-PAM as a prophylactic treatment for OP poisoning.

**Conclusion:**

*In vitro *and *in vivo *data described here demonstrate the potential advantages of rePON1 and BL-3050 for treatment of OP toxicity and chronic cardiovascular diseases like atherosclerosis. The *in vivo *data also suggest that rePON1 and BL-3050 are stable and safe, and could be used for acute, and possibly repeated treatments, with no adverse effects.

## Background

Serum paraoxonase (PON1) is a calcium-dependent lactonase, with lipophilic lactones constituting its primary substrates [1-3]. When associated with HDL, an increase in the stability and lipo-lactonase activity of PON1 were measured both *in vivo *and *in vitro *[4,5]. Also, HDL-PON1 complex inhibits LDL oxidation [6,7], and stimulates cholesterol efflux from macrophages [8]. Previous studies of PON1 showed that knockout mice were highly susceptible to atherosclerosis [9], and serum PON1 levels, and polymorphism, were related to the level of cardiovascular disease [10,11], all of which indicate a role of PON1 for the prevention of atherosclerosis. PON1 also exhibits hydrolytic activity against certain organophosphates (OPs), including the toxic oxon metabolites of a number of insecticides, and nerve agents such as sarin and soman [12,13], and has thus the potential to protect against OP poisoning. Indeed, PON1 knockout mice exhibit a significant increase in sensitivity to diazoxon [14], paraoxon, chlorpyrifos and chlorpyrifos-oxon [9], and the toxic effects can be reversed by administrating rabbit PON1 [15]. Although these properties render PON1 an attractive candidate for the treatment of atherosclerosis, and pesticides or nerve agents toxicity, certain characterizations of human PON1 hamper such uses.

Human PON1 (huPON1), is sensitive to a range of challenges, including the presence of oxidizing agents, glucose, and thiols [16-19]. The complex of HDL (specifically apoA-I), stabilizes the enzyme. Thus, when anchored onto functional HDL-apoA-I, PON1 exhibits anti-atherogenic activity [20], but not in its lipid-free form [21,22]. However, cardiovascular disease (CVD) involves the modification of HDL composition and structure giving rise to "dysfunctional HDL" [23]. HDL-associated enzymes including PON1 become dysfunctional and/or depleted under these conditions, as well as under inflammatory conditions [24], and metabolic diseases such as type 1 and type 2 diabetes [23,25], metabolic syndrome (MetS) [26], and premature CVD [27].

Acute-phase response is also associated with decreased PON1 activity, probably due to the displacement of PON1 from HDL [26]. It appears, therefore, that a highly robust PON1, and perhaps a regeneration of HDL particles, might be needed for therapeutic applications, as demonstrated by the application of apoA-I Milano [28] and apoA-I mimetics [29]. The application of HDL-PON1 complex with improved stability and efficacy as described in this paper might therefore be needed for effective HDL-therapy.

In addition, the catalytic efficiency of huPON1 with most organophosphates, and effectively all highly toxic nerve agents, is not sufficiently high to provide substantial protection [14,30]. In fact, PON1's activity with many OPs is comparable to the weak, promiscuous activity of serum albumin towards these agents [31]. Another limitation of huPON1 is its poor stability and tendency for aggregation [32,33]. This may limit the therapeutic usages of the enzyme in which relatively high concentrations are administered by the intravenous route.

Directed evolution is extensively used to improve protein properties, such as stability, binding affinity, or catalytic efficiency. We have applied directed evolution to generate recombinant PON1 (rePON1) that expresses in a soluble and functional form in *E. coli*, and exhibits enzymatic properties, and HDL binding and stimulation capabilities, that are essentially identical to those of huPON1 [34,35]. The often-used rePON1 variant G3C9 is closest in sequence to rabbit PON1 (94% amino acid identity) and huPON1 (85% identity). RePON1-G3C9 has also provided the basis for the directed evolution of other recombinant variants that exhibit 10 to 380-fold higher catalytic efficiencies with various toxic OPs relative to huPON1 [13,36,37]. The therapeutic potential of *in vitro *evolved proteins, has been convincingly demonstrated with antibodies and antibody fragments [38], but is far less developed with enzymes [39]. Of particular concern is the toxicity of engineered proteins such as rePON1 whose sequence differs from the human protein.

In this study we examined the *in vitro *stability of rePON1 as a purified protein or in a complex with reconstituted HDL (rePON1-HDL), and compared them to huPON1, and huPON1-HDL (huHDL). We used various conditions that mimic physiologically relevant challenges leading to dysfunctional HDL. The results indicate significantly higher stability and reactivity of rePON1 and rePON1-HDL when compared to huPON1 and huHDL, and suggest that rePON1 and rePON1-HDL may exhibit an improved potential for *in vivo *treatments. To further address the issue of *in vivo *applicability, we generated reconstituted complexes of rePON1 with apoA-I and the phospholipids POPC (dubbed BL-3050) and evaluated the potential toxicity of BL-3050 by single administration to mice, and by repeated administrations in the course of two weeks, and observed no adverse effects. Finally, to examine whether BL-3050 is active *in vivo*, we applied an animal model for OP poisoning. BL-3050 and purified rePON1 were administrated few minutes, or 14 hours, prior to chlorpyriphos-oxon (CPO) poisoning to evaluate their protection abilities. Both, BL-3050 and rePON1 showed a significant protective effect *in vivo*. These results indicate that rePON1, and BL-3050, could be applied *in vivo *while taking advantage of the improved stability and catalytic efficiencies of rePON1 variants.

## Methods

### Materials

1-Palmitoyl-2-Oleoyl-Phosphatidyl Choline (POPC) was purchased from Avanti Polar Lipids (Alabaster, AL, USA). Free cholesterol (FC) was from Sigma-Aldrich (Israel). Sodium hypochlorite (NaOCl, 6%) was from Gadot Lab. Supplies (Israel). Bio-Beads SM-2 was from Bio-Rad (Hercules, CA, USA). Chlorpyrifos oxon was from Greyhound Chromatography and Allied Chemicals (Birkenhead, UK). Atropine was from Teva (Israel), and 2-pyridine aldoxime methyl chloride (2-PAM) was purchased from Sigma-Aldrich (Israel). All chemicals were of the highest-purity analytical grade.

### PON1 preparation

Recombinant PON1 variant rePON1-G3C9 (gi: 40850544) carrying a 8xHis tag at the C-terminus, was expressed and purified as described [35]. Re-PON1 was stored in the storage buffer (50 mM Tris pH 8.0, 50 mM NaCl, 1 mM CaCl_2 _and 0.1% tergitol). Human PON1 (192Q) was kindly provided by Prof. Michael Aviram (Technion, Israel) and Dr. Dragomir Draganov (WIL Research Laboratories, Ashland, OH) and was stored with 20% glycerol. The enzyme was purified from pooled blood sera of healthy individuals by three sequential gel chromatographic steps: Cibacron Blue 3GA, DEAE I, and DEAE II as described [40]. The huPON1 sample applied here had a specific arylesterase activity of ~1000 U/mg (1 U = 1 μmol of phenyl acetate hydrolyzed per minute per mg protein at 1 mM substrate concentration), as previously reported [41], and SDS-polyacrylamide gel electrophoresis indicated a single band (≥ 90% protein purity). In all the assays rePON1 and huPON1 (stock concentrations ~1 mg/ml) were similarly diluted in Tris-buffered saline (TBS; 10 mM Tris pH 8.0, 150 mM NaCl) while keeping the same final percentage of tergitol in all samples. All the data represents the mean and S.D. obtained from at least three independent experiments.

### Human HDL preparation

Human HDL (192Q) was kindly provided by Prof. Michael Aviram (Technion, Israel). HDL was derived from normolipidemic human volunteers by discontinuous density gradient ultracenrifugation [42]. The HDL sample was dialyzed against TBS with 1 mM CaCl_2_, and its protein content was determined with the Folin phenol reagent.

### rePON1-HDL preparation for in vitro studies

Discoidal reconstituted HDL (rHDL) containing POPC, FC and apoA-I at a starting molar ratio of 100/5/1, were prepared by the cholate dialysis method as previously described [35]. The homogeneity of the preparations was assessed by non-denaturing gradient gel electrophoresis (4-20% polyacrylamide, Pharmacia) and electron microscopy indicating the formation of particles of ~10 nm [43,44]. RePON1 was freshly delipidated using Bio-Beads, diluted to 0.1 *μ*M in the assay buffer, and incubated with a 100-fold molar excess of rHDL (10 *μ*M of rHDL; 0.6 mg/ml apoA-I).

### PON1 in vitro inactivation by calcium chelation

PON1 and PON1-HDL samples were diluted in TBS with 1 mM CaCl_2 _to a final PON1 concentration of ~0.1 *μ*M. An equal volume of inactivation buffer (4 mM EDTA and 8 mM β-mercaptoethanol in 50 mM Tris, pH 8.0) was added, and samples were incubated at 37°C. At different time points, aliquots of the reaction mixtures were diluted 40-fold in activity buffer (50 mM Tris pH 8.0, 1 mM CaCl_2_), and the residual PON1 activity was determined with 2 mM phenyl acetate. The resultant inactivation rates were fitted to either mono- or double-exponentials [35,45]. PON1 inactivation in the absence of calcium chelator was examined by testing the residual activity after the incubation of PON1 and PON1-HDL samples in phosphate-buffered saline (PBS) supplemented with 1 mM CaCl_2 _for 24 hrs at 37°C.

### PON1 in vitro thermal inactivation

RePON1 and huPON1 (~1 *μ*M) were incubated in TBS with 1 mM CaCl_2 _at a range of temperatures (25 - 80°C) for 30 mins. The samples were briefly cooled on ice, diluted 10-fold in activity buffer, and the residual PON1 activity determined with 2 mM phenyl acetate at 25°C. Data were fitted to a sigmoidal decay function and the apparent mid-melting temperature values (Tm) were derived. Residual activity (%) = 100/(1 + exp(m*(T-Tm)); whereby T is the temperature, and m is a constant. Separate fits for rePON1 and huPON1 were obtained while masking the first point that showed a non-regular decline and corresponded to a loss of ~20% of activity at relatively low temperature (30°C for human PON1, and 40°C for G3C9). The data sets were then normalized to 100% at the lowest temperature, so that the rePON1 and huPON1 plots could be overlayed and fitted. The Tm values were not affected by this normalization.

### PON1 in vitro inactivation by glutathione

PON1 and PON1-HDL samples were incubated at 37°C at PON1 concentrations of ~0.1 *μ*M in TBS supplemented with 1 mM CaCl_2 _and various glutathione (GSH) concentrations (0 - 20 mM), or various GSH/GSSG ratios using a constant total glutathione concentration (GSH plus 2xGSSG) of 10 mM. Following 40 minutes incubation, the reaction mixtures were diluted 40-fold in activity buffer, and residual PON1 activity was determined with 2 mM phenyl acetate.

### PON1 in vitro oxidation by hypochlorite

Hypochlorite oxidation was performed as described [46] with certain modifications. PON1 and PON1-HDL oxidation was performed with NaOCl in 10 mM phosphate buffer (pH 8.0) that was preincubated with 100 *μ*M nitrilotriacetic acid (NTA) for 12 hrs at 25°C (this pre-incubation aimed at chelating the traces of transition metal ions that may enhance the oxidation reactions), and then supplemented with 1 mM CaCl_2_. PON1 (0.1 *μ*M) and HDL (5 *μ*M) samples were incubated with a range of NaOCl concentrations (0 - 200 *μ*M) for 24 hrs at 37°C. The reaction mixtures were subsequently diluted 40-fold in activity buffer, and the residual PON1 activity was determined with 2 mM phenyl acetate. It should be noted that the reproducibility of these oxidation assays was limited, although consistent differences between the recombinant and human PON1 and HDL complexes were observed in all the assays. It appears that these oxidative assays are very sensitive to the presence of the traces of the transition metal ions in the assay buffer. Indeed, pre-incubating the buffer with NTA (100 *μ*M) gave more reproducible results.

### PON1 in vitro oxidation by Tetranitromethane (TNM)

PON1 and PON1-HDL samples were diluted in PBS (~0.1 *μ*M of PON1 and ~5 *μ*M of HDL) supplemented with 1 mM CaCl_2 _and 0.01% tergitol. Inactivation was initiated by addition of TNM (1 mM final concentration) and incubation of the samples at 37°C. At different time points (0, 0.25 and 0.5 hrs), aliquots of the reaction mixtures were taken, diluted 40-fold in activity buffer, and the residual PON1 activity was determined with phenyl acetate as above.

### BL-3050 preparation for in vivo studies

The standard cholate dialysis protocol for the preparation of rHDL [35,43,44] was optimized for large-scale productions of *in vivo *applicable material. RePON1 and human apoA-I [47] were expressed in *E. coli *as described [35]. The proteins were purified to a high degree of homogeneity by fast performance liquid chromatography (FPLC) on a Ni-NTA column, followed by a High Trap Q column (Pharmacia). The endotoxins level of the purified proteins was assayed with Gel Clot kit by BioWhittaker Cambrex. POPC-apoA-I mixtures were prepared by suspending POPC (1 gr) and apoA-I (0.32 gr) in TBS with 2% sodium deoxycholate. The resultant mixture was vigorously vortexed and sonicated until a clear solution was obtained. POPC-apoA-I mixture was then incubated with the freshly delipidated re-PON1 (0.16 gr) to yield rePON1-POPC-apoA-I mixture. The resultant mixture was extensively diluted in TBS to remove sodium cholate, and concentrated by ultrafiltration to yield discoidal rePON1-POPC-apoA-I complexes at the final concentration of the components of 2 mg/ml, 12.5 mg/ml and 4 mg/ml, respectively, in a dosage volume lower than 10 ml/kg.

### In vivo studies

All *in vivo *studies were performed at HBI (Harlan Biotech, Israel) following the review of the Committee for Ethical Conduct in the Care and Use of Laboratory Animals of the Hebrew University, Jerusalem, the Institutional Animal Care and Use Committees (IACUC) responsible for approving HBI animal usage applications and regulations set forth, and in compliance with its respective registration under: NIH accreditation No.: OPR-A01-5011 HU. The ethic approvals for intravenous toxicity in mice (single dose acute toxicity and two-week repeated toxicity) and organophosphate detoxification studies are MD-07-10470-5 and MD-08-11201-5, respectively.

### BL-3050 single dose acute intravenous (IV) toxicity in mice

Potential toxic effects of BL-3050 (60 mg/kg) were examined following a single intravenous injection to the tail vein at a dose volume of 0.2 mL to male and female C57BL/6J mice. Two additional groups comprised of TBS or POPC were evaluated under identical experimental conditions and served as the control groups. All groups comprised *n *= *6 *mice/group (3 males and 3 females) excluding TBS group which consisted of *n *= *4 *mice/group (2 males and 2 females). All animals were closely observed for signs of adverse effects and toxicity during the first 24 hours and were sacrificed after 4 days.

### BL-3050 two-week repeated intravenous (IV) toxicity in mice

Repeat dose toxicity of BL-3050 (60 mg/kg) was assessed following 7 intravenous injections to the tail vein at a dose volume of 0.2 mL to male & female C57BL/6J mice, carried out every other day during the entire two-week study period. Two additional groups comprised of TBS or POPC were evaluated under identical experimental conditions and served as the control groups. All groups comprised of *n *= *10 *mice/group (5 males and 5 females). All animals were observed closely for signs of adverse effects during the two-week study period until animals were sacrificed.

All animals from both studies (single dose acute toxicity and two-week repeated toxicity) were subjected to a full detailed necropsy and gross pathological examination at termination time. Furthermore, selected target organs/tissues (heart, lung, kidney, spleen, liver) were harvested from all the animals in the study during the scheduled necropsy, weighed wet as soon as possible following their dissection and fixed in 10% neutral buffered formalin (approximately 4% formaldehyde solution) for at least 48-hr fixation period prior to their analysis. Tissues were trimmed, embedded in paraffin, sectioned at approximately 5 microns thickness and stained with Hematoxylin & Eosin (H&E) for histopathological analysis.

### Organophosphate detoxification in the animal model

Organophosphate (OP) intoxication was induced by a single oral gavage (PO) administration of chlorpyrifos-oxon (CPO) to male C57BL/6J mice (n = 8) at a dose of 23 mg/kg at a dose volume of 10 mL/kg (23 mg/kg was determined as the LD_50 _in preliminary studies; oral gavage was chosen for safety reasons as it minimizes the risk of leakage from the skin and contamination). The study consisted of the following groups: untreated (where only CPO was administrated), TBS (buffer only; 0.2 mL), POPC (2.5 mg/animal), rePON1 (0.4 mg and 0.63 mg/animal), BL-3050 (0.4 mg rePON1/animal), and atropine (20 mg/kg) with 2-PAM (25 mg/kg). TBS, POPC, rePON1 and BL-3050 were injected intravenously to the tail vein at a dose volume of 0.2 mL to mice, and atropine with 2-PAM were injected to the intraperitoneal (IP) into the posterior abdominal regions at the dose volume of 10 mL/kg. Treatments with the test article or control were carried out 5 minutes, 3 hr or 14 hours prior to induction of CPO intoxication as described in Table 1. All animals were observed closely for clinical signs following CPO intoxication during the first 24 hours before they were sacrificed.

**Table 1:** representative results of detoxification studies

Time prior CPO induction	Group^1^	Score	Mortality (%)	Severe reaction (%)
				

	untreated^2^	3.6	62.5	37.5

	TBS	3.8	75	25

	POPC	3.9	87.5	12.5

				

5 min				

	Atropine+2PAM^3^	0.5*	0	0

	BL-3050^4^	1.3*	12.5	0

				

3 hours				

	Atropine+2PAM^3^	2.1	25	12.5

	BL-3050^4^	1.3*	12.5	0

	rePON1-TBS^4^	1.4*	12.5	12.5

				

14 hours				

	BL-3050^4^	3.0	50	25

	rePON1-TBS^4^	1.8*	0	12.5

	rePON1-TBS^5^	1.1*	12.5	12.5

Single oral gavage of CPO (23 mg/kg, 10 mL/kg dose) to male C57BL/6J mice was preceded (5 min, 3 hr or 14 hrs prior to CPO intoxication) by no treatment or by injection of TBS (buffer only), POPC (2.5 mg/animal), rePON1 (0.4 mg and 0.63 mg/animal), BL-3050 (0.4 mg rePON1/animal), and atropine (20 mg/kg) with 2-PAM (25 mg/kg). Animals were observed closely for clinical signs during the first 24 hours following CPO intoxication.

* - Significant difference (p < 0.05) compared to all control groups using Mann-Whitney test (see Methods and Results and Discussion sections for more details).

1 - All groups comprised of 8 mice

2 - Mice were only poisoned by CPO

3 - Atropine and 2-PAM concentrations were 20 mg/kg and 25 mg/kg, respectively

4 - Protein estimation amount for rePON1 was 0.4 mg/animal.

5 - Protein estimation amount for rePON1 was 0.63 mg/animal.

All clinical signs noted following CPO intoxication were categorized to mild, moderate or severe reactions. Mild reactions were characterized by straub tail and/or ataxia and/or diarrhea. Moderate reactions consisted in addition decreased motor activity and/or dyspnea while animals with severe reactions exhibited in addition ventral position and/or tremors as well. The overall reactions observed following CPO intoxication were scored using semi-quantitative grading of five grades (0-4), taking into consideration the severity of the reactions (0 = No Reactions, 1 = Mild Reactions, 2 = Moderate Reactions, 3 = Severe Reactions, 4 = Mortality).

## Results and Discussion

### Higher stability of rePON1 and rePON1-HDL

**Figure 1:**
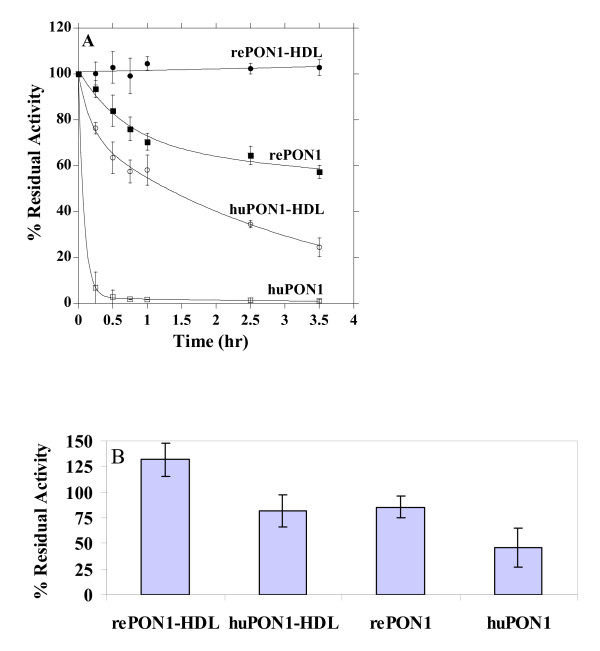
**Inactivation of the recombinant and human PON1, and PON1-HDL complexes in the presence of calcium chelator (A) or in buffer (B)**. Data represents mean ± SD of at least 3 independent experiments. **A**. Inactivation kinetics of rePON1, rePON1-HDL, huPON1, and huPON1-HDL (huHDL) by EDTA (4 mM) and β-mercaptoethanol (8 mM) at 37°C. Residual activity at various time points was determined by initial rates of phenyl acetate hydrolysis and plotted as percent of the activity at time zero. Data were fitted to a single exponential for rePON1-HDL, and to double-exponentials for the remaining samples [35]. **B**. Inactivation of rePON1, rePON1-HDL, huPON1, and huPON1-HDL (huHDL), in PBS buffer with 1 mM CaCl_2_. Residual PON1 activity after 24 hrs incubation at 37°C is presented as percentage of the initial activity.

PON1 possess two calcium atoms - a highly buried "structural calcium" that shows high affinity, and the lower-affinity active-site "catalytic calcium" [48,49]. The depletion of calcium by chelators, or even by dilution in buffers with no calcium, renders PON1 catalytically inactive by removing the catalytic calcium, and eventually leads to loss of the structural calcium and irreversible denaturation of the enzyme [50]. Calcium affinity is correlated with the overall stability of PON1 [49,50], and may therefore reflect the half-life of the enzyme in serum, and/or when stored. RePON1 exhibits higher calcium affinity, possibly by virtue of its closer similarity to rabbit PON1 [49,50], and higher stability altogether, by virtue of the improved packing of its hydrophobic core mediated by the mutations acquired in the directed evolution process [51]. Indeed, a test of inactivation rates in the presence of EDTA as chelator (Figure 1a) [35], indicated an almost immediate inactivation of huPON1 (*t*_1/2 _= 5 min), in oppose to slow inactivation of rePON1 (*t*_1/2_~5 hrs). The association with HDL greatly increases the enzyme's stability, as previously reported [35,45]. Nonetheless, huHDL shows rather limited stability (*t*_1/2 _= 1.3 hrs). In contrast, the complex of rePON1 with *in vitro *reconstituted HDL particles shows no inactivation within the timescales examined here, and only 21% inactivation after 24 hrs (*t*_1/2 _= 70 hrs) [35].

**Figure 2:**
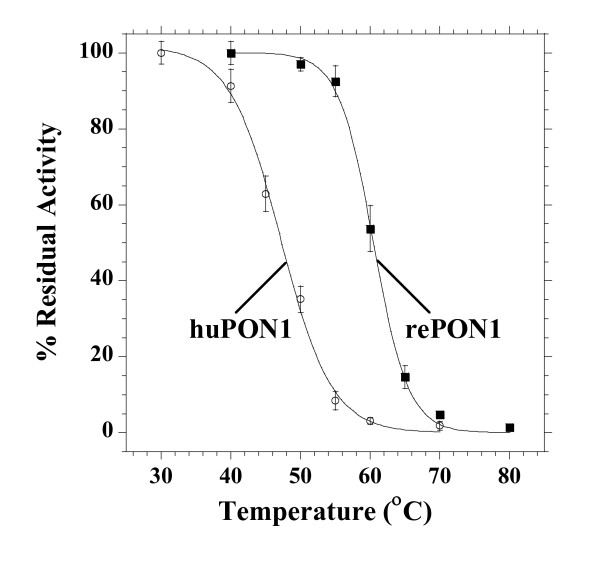
**Heat inactivation of rePON1 and huPON1**. Purified enzymes diluted in TBS with 1 mM CaCl_2 _were incubated at the range of temperatures (25 - 80°C) for 30 mins, and residual PON1 activity was determined. Data were fitted to the sigmoidal decay function to give Tm values of 47.2°C for huPON1 and 60.4°C for rePON1 (the corresponding *m *values are 0.273 and 0.399; see Methods). Each point represents mean ± SD of 3 experiments.

The higher stability of rePON1 was also apparent in the absence of specific calcium chelators: huPON1 exhibited 54% inactivation when incubated in phosphate buffer containing 1 mM CaCl_2 _for 24 hrs, whereas rePON1 remained intact (Figure 1b). Inactivation of huPON1 after dilution in PBS was observed in other studies [16,19]. Indeed, huPON1-HDL only preserved its activity in phosphate buffer suggesting that the HDL stimulation was counterbalanced by loss of activity due to calcium loss. In contrast, rePON1 gained ~30% arylesterase activity due to enzymatic stimulation by HDL. Although phosphate interacts with calcium, its ligating affinity (6.3 × 10^-3 ^M) [52] is far lower than huPON1's (3.6 × 10^-7 ^M) [49]. Hence, the major inactivating force acting in this experiment is the dilution of lipids, and loss of conformational stability. The improved conformational stability of rePON1 was also apparent in the results of the standard test of protein stability by thermal denaturation: rePON1 shows >13°C higher melting temperature than huPON1 (Figure 2). The higher conformational stability of rePON1 may give it higher resistance against the cumulative damage of different challenges that inactivate PON1 and HDL in vivo, as suggested by the results presented below.

### Inactivation by physiologically-related challenges

**Figure 3:**
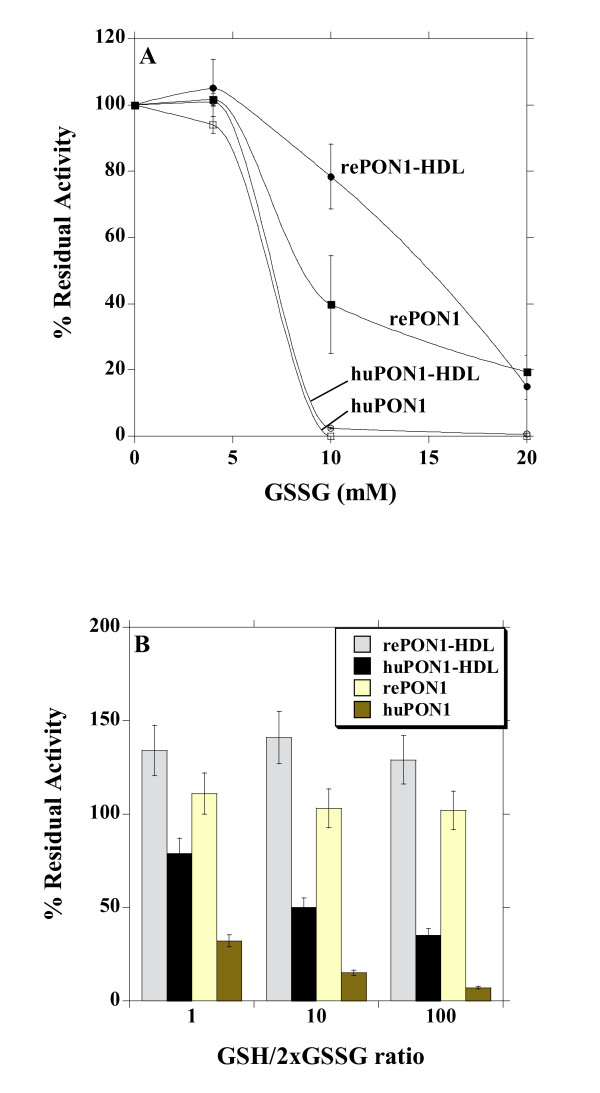
**Inactivation of the recombinant and human PON1, and PON1-HDL complexes by glutathione**. Data represents mean ± SD of at least 3 independent experiments. **A**. Recombinant and human PON1, and the corresponding HDL complexes, were incubated with increasing concentrations of GSSG at 37°C. Residual PON1 activity was determined after 40 min of incubation, and plotted as percentage of the initial activity. **B**. Recombinant and human PON1, and the corresponding HDL complexes, were incubated with various GSH/GSSG ratios (the ratios are expressed as the ratio of glutathione equivalents, i.e., the ratio of GSH to 2xGSSG) whilst keeping the total glutathione concentration at 10 mM. Residual PON1 activity was determined after 40 min of incubation, and plotted as percentage of the initial activity.

We tested several reagents that have been shown to inactivate PON1 in relation with various physiological stresses that induce HDL and/or PON1 inactivation *in vivo*. The glutathione redox couple is present in mammalian cells in concentrations between 1 and 10 mM, with the reduced form (GSH) predominating the oxidized one (GSSG) [53]. Glutathione, in both its reduced and oxidized forms, was shown to mediate PON1 inactivation, possibly by reacting with PON1's disulphide bond, or free cysteine [18,54]. Indeed, oxidative stress is characterized by changes in glutathione concentrations and decreased reduced/oxidized ratios (GSH/GSSG) in the range of 10 to 1 [55]. In healthy humans, plasma glutathione is present at ~1 mM concentrations, while enhanced protein glutathiolation is associated with increased oxidative stress [56]. The increased GSH and GSSG concentrations applied here for short incubations (40 mins), may also reflect damages that occur *in vivo *at lower concentration yet on much longer time scales (weeks, or months). Indeed, GSSG was shown to induce the loss of PON1 and HDL activity (including the ability to mediate macrophage cholesterol efflux) in a dose-dependent manner [18]. Similar results were obtained here for huPON1, although rePON1 showed much lower sensitivity under these conditions (Figure 3). The huPON1-HDL complex shows essentially the same inactivation rate as huPON1, but the reconstituted rePON1-HDL complex does provide improved protection against oxidized glutathione (Figure 3a). At 10 mM GSSG, huPON1 and huHDL totally lost their activity, whereas significantly higher activity was retained in the rePON1 and rePON1-HDL samples (40% and 80%, respectively). A mixture of reduced and oxidized glutathione at 10-100 ratio (10 mM total glutathione), induced human PON1 and huHDL inactivation at a higher rate than oxidized glutathione on its own. Nonetheless, rePON1 and rePON1-HDL proved highly resistant against the effects of both reduced and oxidized glutathione, and their activity remained intact at all the GSH/GSSG ratios tested (Figure 3b).

Hypochlorite was used to mimic oxidative damages to HDL that undermine its composition and structural stability [46]. It is one of the products of myeloperoxidase (MPO), a heme enzyme expressed by macrophages in atherosclerotic lesions, and is thought to be a key mediator of lipoprotein oxidation [57]. Physiological concentrations of HOCl have not been accurately determined, although some reports suggest that, at sites of acute inflammation, the molar ratio of oxidant to HDL may be as high as 30:1 [46,58]. HDL contains both a protein and lipid component, either of which may be oxidatively damaged during atherosclerosis [59,60].

**Figure 4:**
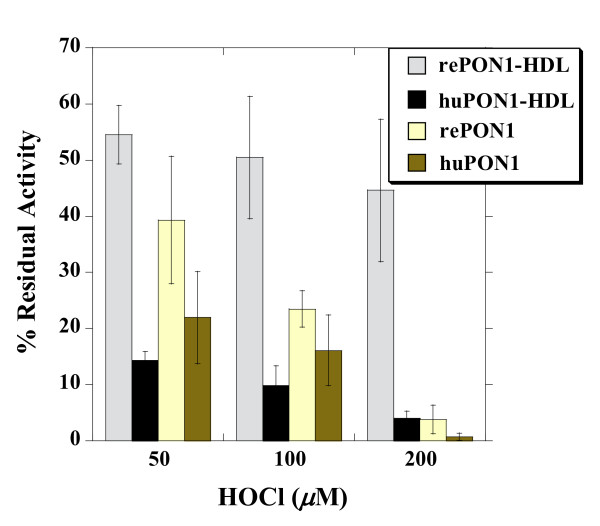
**Inactivation of the recombinant and human PON1, and PON1-HDL complexes by hypochlorite**. Recombinant and human PON1, and the corresponding HDL complexes, were incubated with increasing concentrations of hypochlorite at 37°C. Residual PON1 activity was determined after 24 hrs incubation, and plotted as percentage of the initial activity. Residual PON1 activity in the absence of hypochlorite remained largely unchanged for all the samples (≥ 75%). Data represents mean ± SD of 4 independent experiments.

Oxidation by hypochlorite led to the dose-dependent inactivation of PON1 on its own and in complex with HDL (Figure 4). RePON1-HDL complex showed a significantly higher resistance (> 10-fold) to hypochlorite inactivation compared to huHDL (45% and 4% residual PON1 activity at 200 *μ*M HOCl, respectively). In the absence of HDL, both rePON1 and huPON1 showed increased sensitivity to hypochlorite inactivation. However, rePON1 was approximately 2-fold more resistant to this oxidative damage than huPON1.

**Figure 5:**
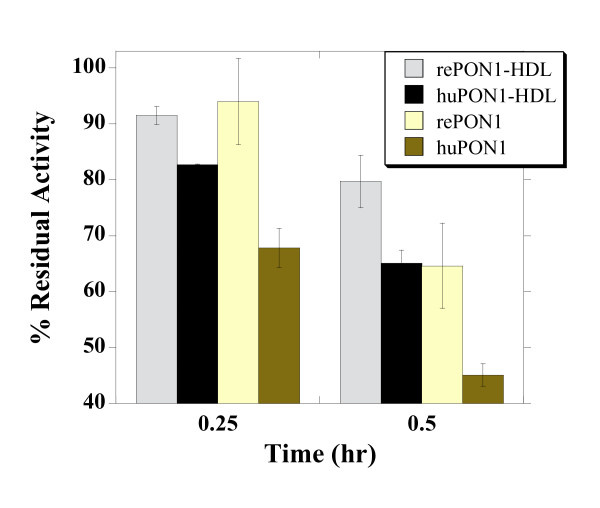
**Inactivation of recombinant and human PON1, and PON1-HDL complexes by tetranitromethane**. PON1 samples were incubated with 1 mM of TNM at 37°C. Residual PON1 activity was determined after 0.25 and 0.5 hrs of incubation, and plotted as percentage of the initial activity. Residual PON1 activity with no addition of TNM remained unchanged for all the samples (~100%). Data represents mean ± SD of 3 independent experiments performed in duplicates.

Tetranitromethane (TNM) that leads to nitration of tyrosine, cysteine, and methionine residues in proteins, can also be used to mimic PON1 and HDL oxidation during oxidative stress [60,61]. Indeed, HDL isolated from patients with cardiovascular disease contains elevated levels of 3-chlorotyrosine and 3-nitrotyrosine, that are two characteristic products of MPO [62]. Oxidation by TNM led to the time-dependent inactivation of PON1, on its own and in complex with HDL (Figure 5). RePON1-HDL complex showed a higher resistance to TMN inactivation compared to huHDL (80% and 65% residual PON1 activity after 0.5 hr of incubation, respectively). In the absence of HDL, both rePON1 and huPON1 showed increased sensitivity to TMN inactivation. However, rePON1 was approximately 1.5-fold more resistant to this oxidative damage than huPON1 (65% and 45% residual PON1 activity after 0.5 hr of incubation, respectively). Inactivation was specific to TNM treatment, and no loss of PON1 activity was observed in control samples containing no TNM.

Tests of other physiologically relevant agents did not yield sufficiently meaningful, and/or reproducible, results. Although PON1 was shown to be inactivated in the presence of glucose, possibly due to the glycation of lysine residues [16], we observed no specific inactivation by glucose (up to 100 mM). It appears that the glucose-mediated effects are masked by the rapid inactivation of huPON1 due to its dilution into buffer (Figure 1b), whereas rePON1 is relatively resistant to the dilution effect, as well as to the effects of glucose. Oxidative agents such as hydrogen peroxide, or tert-butyl peroxide, failed to induce specific inactivation (i.e., inactivation beyond the levels of buffer alone) under the conditions applied here. Competing PON1 binding to rHDL by serum amyloid A indicated that rePON1 remained fully associated with HDL in presence of the highest concentrations tested (2 mg/ml). In contrast, huPON1 was readily displaced from HDL and thereby became inactivated (data not shown). However, the observed inactivation of huPON1 may in part be ascribed to loss of activity following long incubations in buffer as described above.

### BL-3050 toxicity in mice

To assess the potential toxic effects of BL-3050, a single intravenous injection (IV) to C57BL/6J mice was performed (acute study) and the effects were compared to the vehicle control groups of TBS, and TBS with POPC. All animals were observed closely for signs of adverse effects (see Methods), and were subjected to a full necropsy on termination day. The results indicated no toxic effects, or adverse immune responses, as indicated by the histopathological analysis. We subsequently assessed the potential toxic effects of BL-3050 with respect to its intended use as treatment for atherosclerosis. Repeated IV injections to C57BL/6J mice were carried out every other day during a period of two weeks. Two equally sized groups, subjected to either TBS, or POPC, under identical experimental conditions served as control groups. All groups comprised 10 mice (5 males and 5 females). Animals were observed closely for signs of adverse effects and/or mortality during the 14-day injection period. The results indicated no mortality, or any other conspicuous treatment-related adverse reactions, in any of the tested animals throughout this period. No gross pathological findings were evident macroscopically among the injected animals supported by histopathological analysis and no changes in mean group organ weight values were observed. These results suggest that BL-3050 injected intravenously (IV) every other day during two weeks can be considered a safe treatment.

### *In vivo *efficacy of rePON1 and BL-3050

We examined the possibility of *in vivo *treatment with rePON1, and BL-3050 (reconstituted complexes of rePON1, apoA-I and POPC) using mice as a model. HDL-like particles were produced from recombinant apoA-I [47] and POPC. The standard cholate dialysis protocol [35,43,44] was optimized towards large-scale production of *in vivo *applicable material, while excluding cholesterol from this composition aimed at potential therapy of CVD. The injected amounts of apoA-I-POPC were selected to match the amounts applied with apoA-I Milano in patients trials [63], and PON1 was added to the complex at 1:2 molar ratio relative to apoA-I. Although higher ratios (≥ 1:20) can induce higher stability and stimulation [35], these would have dictated impossibly high amounts of apoA-I-POPC. In addition, *ex vivo *studies indicated that 1:2 PON1:apoA-I ratios are sufficient to induce marked effects of cholesterol macrophage efflux and LDL oxidation [21]. The concentrations of rePON1, and rePON1-HDL, were adjusted to fit a volume dosage of 10 mL/kg.

As a preliminary measure of *in vivo *applicability, we applied the widely used chlorpyrifos-oxon (CPO) toxicity model [15]. CPO intoxication was induced by administration of CPO at a dose level of 23 mg/kg (10 mL/kg) by single oral gavage to male C57BL/6J mice. The selection of 23 mg/kg dose was based on preliminary CPO studies where this dose was determined as the LD_50 _(data not shown). This dose induced mortality incidence of ≥ 50%, and all the surviving animals exhibited adverse effects. Groups of 8 male C57BL/6J mice were treated with various test items followed by intoxication with CPO. Atropine (20 mg/kg) *plus *2-PAM (25 mg/kg) served as a control treatment for the prevention of OP toxicity [64]. Untreated mice, and control mice treated with TBS buffer or POPC, showed poor protection where all mice in these groups either died or showed sever reaction and the clinical score was 3.6-3.9 (Table 1). In contrast, the administration of BL-3050 or rePON1-TBS reduced dramatically the mortality and severe reactions to 12.5% when given 3 hours prior to poisoning with score of 1.3-1.4, and 0-12.5% when rePON1-TBS was given 14 hours prior to poisoning (score 1.1-1.8). Strikingly, when rePON1 was administered at a higher dose, better protection overall was observed (score 1.1 versus 1.8) when 62.5% of the mice had no reaction (data not shown). In contrast to BL-3050 and rePON1-TBS, the protection provided by atropine *plus *2-PAM decreased as the time gap between the treatment and OP poisoning increased (score 0.5 versus 2.1). Indeed, prophylactic activities of rePON1 were observed following IV administrations 5 minutes up to 14 hours prior to the OP exposure, with optimal effects after 3 hours. However, for reason that are unclear at present, at the longest duration of pre-treatment (14 hrs), the protection by rePON1 on its own was higher than for its HDL complex (BL-3050). The latter provided only a limited protection (scores of 1.1-1.8 and 3.0, respectively). One possibility is, that upon the IV administration, rePON1 can associate with endogenous HDL particles that may stabilize it and slow its clearance rate. Further pharmacokinetic studies may determine the rate of clearance in blood, and shed light on the observed differences.

## Conclusion

This study shows the highly improved stability and activity of the engineered rePON1, and of its HDL complex (rePON1-HDL and BL-3050), and their applicability *in vivo*. RePON1 and BL-3050 are significantly more stable than their human counterparts. Whilst the conditions applied here only partly mimic the *in vivo *challenges, and some conditions such as calcium chelators might not be relevant *in vivo*, the fact that with the entire range of challenges tested here rePON1 was found to be more resistant than huPON1, suggests that rePON1 would also be more resistant against physiological challenges that relate to the formation of dysfunctional HDL during atherosclerotic and cardiovascular diseases [23]. The improved stability of rePON1 might also be advantageous for long-term storage, thus increasing the shelf-life of rePON1 based formulations.

Furthermore, rePON1 described here was generated by directed evolution for bacterial expression, and since it differs from both human and mouse PON1s, it could have created adverse immune responses when repeatedly administrated. Yet, acute and repeated toxicology studies suggested that rePON1 is a non toxic protein, and that BL-3050 administrated IV is a safe particle that can be used for acute treatment with no adverse effects and may also be considered for repeated injections. The *in vivo *activity of the improved rePON1 was demonstrated by using BL-3050 and rePON1 for organophosphate poisoning protection compared to standard treatment of atropine and 2-PAM. The significant prophylactic effect was demonstrated by rePON1 persisting at least 14 hours after its administration. Furthermore, rePON1-TBS results for 14 hours may suggest a dose response where higher amount of rePON1 provided a better protection. Also, these results suggest that rePON1 and BL-3050 can be applied *in vivo *while taking advantage of the improved stability and catalytic efficiencies of rePON1 variant.

Notably, rePON1 administered here significantly differs from the endogenous mouse PON1 (17% amino acid divergence), similar to the case with human PON1 (15% amino acid divergence). These results open the road for the use of rePON1 variants that were evolved for improved OP hydrolysis and can detoxify several common agents that huPON1 cannot [13,36,37]. Since the efficacy of rePON1 and BL-3050 against OP poisoning was similar, and because the phosphotriesterase activity of rePON1 is barely stimulated by HDL, it seems that rePON1 can be used as a detoxifying agent both in its free and POPC-complexed form. On the other hand, the significant (>20-fold) activation of the lactonase and antiatherogenic activities of PON1 by reHDL [21,35] suggests that rePON1-HDL complexes may also exhibit promising antiatherogenic activities *in vivo*.

## List of abbreviations

**apoA-I**: apolipoprotein A-I; **CPO**: chlorpyrifos oxon; **OP**: organophosphate; **Tm**: melting temperature; **CVD**: cardiovascular disease; **FC**: free cholesterol; **GSH**: reduced glutathione; **GSSG**: oxidized glutathione; **HOCl**: hypochlorite; **MetS**: metabolic syndrome; **MPO**: myeloperoxidase; **NTA**: nitrilotriacetic acid; **2-PAM**: 2-pyridine aldoxime methyl chloride; **POPC**: 1-Palmitoyl-2-Oleoyl-Phosphatidyl Choline; **PON1**: serum paraoxonase; **huPON1**: human PON1; **rePON1**: recombinant PON1; **rePON1-TBS**: recombinant PON1 diluted in TBS; **HDL**: high density lipoprotein; **huPON1-HDL **or **huHDL**: human HDL; **rHDL**: reconstituted HDL; **rePON1-HDL**: reconstituted complexes of rePON1 and rHDL; **BL-3050**: reconstituted complexes of rePON1, apoA-I and POPC; **TBS**: Tris-buffered saline; **TNM**: tetranitromethane.

## Competing interests

Recombinant PON1-G3C9 is patented by the Weizmann Institute of Science and the authors LG and DST are inventors. The patents have been licensed to BioLine Innovations Jerusalem, LP for the development of anti-atherogenic and detoxification therapies based on recombinant PON1.

## Authors' contributions

LG, DST and ELN designed the study. LG and SY performed *in vitro *experiments. DB, EN, OK and RT performed *in vivo *experiments. LG, DST and ELN analyzed the data. LG, DST and ELN contributed to writing the paper. All authors read and approved the final manuscript.

## Pre-publication history

The pre-publication history for this paper can be accessed here:

http://www.biomedcentral.com/1472-6904/9/18/prepub
